# Association Between Dietary Patterns and Lifestyle Habits with Vascular Inflammatory Responses in Individuals with Hypertension Living in PM_2.5_-Polluted Areas: A Cross-Sectional Pilot Study in Chiang Mai Province, Thailand

**DOI:** 10.3390/diseases13080258

**Published:** 2025-08-13

**Authors:** Wason Parklak, Kanokwan Kulprachakarn, Sawaeng Kawichai, Puriwat Fakfum, Putita Jiraya, Praporn Kijkuokool, Wiritphon Khiaolaongam, Hataichanok Chuljerm

**Affiliations:** 1 Research Center for Non-Infectious Diseases and Environmental Health, Research Institute for Health Sciences, Chiang Mai University, Chiang Mai 50200, Thailand; wason.p@cmu.ac.th (W.P.); kanokwan.kul@cmu.ac.th (K.K.); sawaeng.kaw@cmu.ac.th (S.K.); nuengpuriwat@gmail.com (P.F.); putita_jiraya@cmu.ac.th (P.J.); 2 School of Health Sciences Research, Research Institute for Health Sciences, Chiang Mai University, Chiang Mai 50200, Thailand; praporn_k@cmu.ac.th (P.K.); wiritphon_k@cmu.ac.th (W.K.)

**Keywords:** air pollution, PM_2.5_, hypertension, cardiovascular disease, vascular inflammation, nutrient intake, lifestyle behaviors, rural area, peri-urban area

## Abstract

Background/Objectives: Exposure to fine particulate matter (PM_2.5_) is linked to increased cardiovascular risk, particularly in individuals with hypertension. This study examined the association between dietary patterns, lifestyle factors, and vascular inflammation among individuals with hypertension living in rural and peri-urban areas of Chiang Mai Province, Thailand. Methods: A cross-sectional pilot study was conducted among 47 participants (23 rural, 24 peri-urban). Data on dietary intake, smoking, alcohol use, anthropometry, and blood chemistry were collected. Serum intercellular adhesion molecule-1 (ICAM-1), vascular cell adhesion molecule-1 (VCAM-1), and interleukin-6 (IL-6) were measured. Partial correlation analysis was used to examine associations with lifestyle factors, adjusting for relevant covariates. Results: Peri-urban participants had significantly higher levels of ICAM-1 [83.0 vs. 50.1 ng/mL], VCAM-1 [639.3 vs. 376.5 ng/mL], and IL-6 [4.80 vs. 1.02 pg/mL] compared to rural participants. Rural individuals reported higher intakes of antioxidant-related nutrients (selenium, β-carotene, niacin, vitamins A, B6, and C), while peri-urban individuals had higher sugar intake. Sugar intake was positively associated with ICAM-1 and VCAM-1, whereas selenium and vitamin C were inversely associated with both ICAM-1 and VCAM-1, while vitamin B6 was inversely associated with VCAM-1 only. Although rural participants had a higher rate of current smoking (34.8% vs. 4.4%), smoking and alcohol use were not significantly associated with inflammatory markers. Conclusion: Rural dietary patterns may be linked to reduced vascular adhesion molecule levels. Further studies with larger samples are warranted to clarify these associations and guide lifestyle strategies for managing vascular inflammation in PM_2.5_-exposed individuals with hypertension.

## 1. Introduction

Cardiovascular disease (CVD) remains the leading cause of morbidity and mortality globally, and presents a substantial burden on healthcare systems [1]. Hypertension is a major risk factor that contributes to the pathogenesis of CVD through multiple mechanisms, including endothelial dysfunction, vascular inflammation, oxidative stress, and structural remodeling [2]. An early indicator of hypertension and atherosclerosis is endothelial dysfunction, which is defined by diminished nitric oxide bioavailability and elevated vasoconstrictor activity [2]. It induces platelet activation, leukocyte adhesion, and vascular stiffening [2]. Elevated shear forces in hypertension exacerbate these processes by stimulating the release of reactive oxygen species (ROS) and activating transcriptional pathways, including nuclear factor kappa-B (NF-κB), which induce pro-inflammatory signaling and upregulate inflammatory mediators [3].

Central to this inflammatory cascade are cytokines including interleukin (IL)-6 and tumor necrosis factor-alpha (TNF-α), which are elevated in individuals with hypertension [4,5]. These cytokines stimulate acute phase responses and contribute to vascular remodeling and fibrosis in cardiovascular tissues [6]. Furthermore, the upregulation of endothelial adhesion molecules, including intercellular adhesion molecule-1 (ICAM-1) and vascular cell adhesion molecule-1 (VCAM-1), enables leukocyte adhesion and transmigration into the vascular intima, key early events in atherogenesis [7,8]. Multiple studies have demonstrated that elevated levels of IL-6, ICAM-1, and VCAM-1 are independently associated with incident hypertension, progression of atherosclerosis, and increased cardiovascular events [9,10].

Air pollution has increasingly been recognized as a significant contributor to the development of various chronic diseases [11,12,13]. Fine particulate matter (PM_2.5_), a major component of air pollution, is a well-established environmental hazard linked to CVD and hypertension through both systematic review and mechanistic studies [11,12,13,14,15]. In Northern Thailand, where PM_2.5_ frequently exceeds safe thresholds, a cross-correlation was observed between increased pollutant levels and hypertension mortality two years later (*ρ* = 0.73, *p* = 0.027) [16]. Additionally, ambient PM_2.5_ exposure accounts for approximately 0.06% of cardiopulmonary deaths in the region [17]. Mechanistically, PM_2.5_ penetrates deep into the respiratory tract and enters systemic circulation, where it interacts with vascular endothelial cells and promotes the overproduction of ROS, triggering oxidative stress. This redox imbalance activates inflammatory signaling cascades, including NF-κB and MAPK pathways, which subsequently upregulate pro-inflammatory cytokines (IL-1β, IL-6, TNF-α) and adhesion molecules (ICAM-1, VCAM-1), contributing to endothelial dysfunction and leukocyte adhesion. Moreover, PM_2.5_ exposure has been shown to induce epigenetic alterations such as DNA hypomethylation (LINE-1, CD40LG, ACE), histone modifications (H3K9me3, H3K27me3), and dysregulation of non-coding RNAs (miR-1, miR-145, lncRNA PEAMIR) which modulate gene expression linked to inflammation, oxidative stress, vascular tone, and coagulation [15,18]. Additionally, murine studies further confirm that chronic PM_2.5_ exposure increases IL-6, ICAM-1, and VCAM-1 levels, accelerating vascular stiffening, plaque formation, and cerebrovascular narrowing [19,20].

Unhealthy lifestyle behaviors including physical inactivity, smoking, alcohol consumption, and poor dietary patterns are recognized as key modifiable risk factors for CVD [21]. Among these, diet plays a central role in shaping cardiovascular health [22]. Pant et al. (2024), and Angelico and Baratta (2023) have consistently linked increased risks of hypertension, dyslipidemia, and systemic inflammation to diets that are high in saturated fats, cholesterol, refined sugars, and sodium [23,24]. These factors all contribute to the development of atherosclerosis and other CVD outcomes [23,24]. Furthermore, dietary patterns have shifted due to the influence of Western food culture, particularly the increased consumption of fast food. Such dietary transitions have been associated with a higher prevalence of obesity and other chronic diseases. This shift has contributed to the gradual displacement of traditional dietary practices among local populations [25]. Conversely, dietary patterns rich in fruits, vegetables, whole grains, plant-based proteins, and unsaturated fats, such as those promoted by the Mediterranean or the Dietary Approaches to Stop Hypertension (DASH) diet, have demonstrated protective effects against CVD by improving lipid profiles [24], reducing oxidative stress [26], and lowering circulating inflammatory markers such as IL-6 and C-reactive protein (CRP) [26,27]. These anti-inflammatory effects may be particularly relevant for individuals exposed to environmental stressors like air pollution.

In Thailand and other low-to-middle-income countries, rapid urbanization has led to marked differences in lifestyle behaviors between rural and peri-urban populations. Individuals in peri-urban communities, particularly those adjacent to metropolitan zones, are more likely to adopt urbanized dietary patterns characterized by increased consumption of convenience foods, sugary beverages, and lower intake of fiber and antioxidants, in contrast to more traditional diets seen in rural areas [28,29]. Therefore, this study aimed to examine whether lifestyle and dietary patterns differ between individuals with hypertension residing in rural and peri-urban areas of Chiang Mai Province both affected by ambient PM_2.5_ and to investigate how these differences are associated with vascular inflammatory markers.

## 2. Materials and Methods

### 2.1. Human Ethics Approval

This study was submitted for ethical review and approval to the Human Experimentation Committee (HEC), Research Institute for Health Sciences, Chiang Mai University, on 19 December 2023. The study was approved and granted ethical clearance on 29 May 2024 (approval no. 30/2024). All participants were fully informed about the study objectives and procedures, and written informed consent was obtained from each participant prior to their enrollment in the study.

### 2.2. Study Locations and Ambient PM_2.5_ Monitoring Data

**Figure 1:**
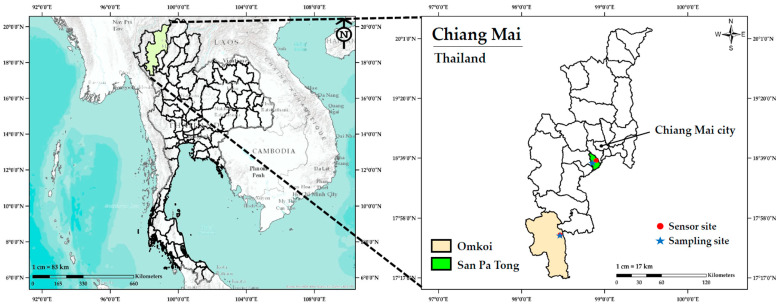
Study locations and monitoring sites for ambient PM_2.5_ concentration and participant recruitment in Chiang Mai Province, Thailand.

This study was conducted in two districts of Chiang Mai Province, Northern Thailand, representing different levels of urbanization: Omkoi (rural) and San Pa Tong (peri-urban). The rural site, Omkoi District, is a remote mountainous area located approximately 206 km southwest of Chiang Mai city. The PM_2.5_ sensor and participant screening site were located at Ban Yang Piang Subdistrict Health Promoting Hospital in Yang Piang Subdistrict, Omkoi District. This region is characterized by forested highlands, limited urban infrastructure, and predominantly subsistence agricultural activities. The peri-urban site, San Pa Tong District, is situated approximately 34 km southwest of Chiang Mai city. The PM_2.5_ sensor was installed at the San Pa Tong Subdistrict Municipality, while participant recruitment and screening were conducted at the Ban Hua Rin Subdistrict Health Promoting Hospital in Thung Satok Subdistrict, San Pa Tong District. This area is classified as a peri-urban zone with moderate population density and ongoing suburban expansion. The geographic locations of the study areas and the data collection sites are illustrated in Figure 1.

Ambient PM_2.5_ concentrations were monitored using PMS7003 sensors manufactured by Beijing Plantower Co., Ltd., Beijing, China. These sensors were assembled into final monitoring units and calibrated for relative humidity adjustment by Nanogeneration Co., Ltd., Chiang Mai, Thailand. PM_2.5_ data were recorded daily and retrieved from the Northern Thailand Air Quality and Health Index (NTAQHI) system [https://www2.ntaqhi.info/ (accessed on 28 October 2024)], managed by the Environmental and Occupational Health Sciences Unit, Research Institute for Health Sciences (RIHES), Chiang Mai University.

**Figure 2:**
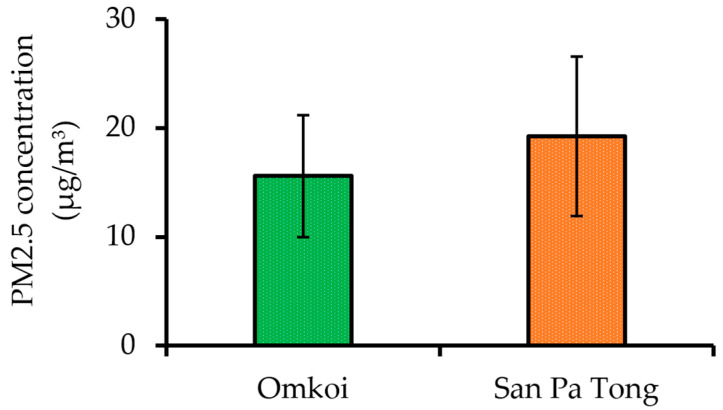
Annual mean PM_2.5_ concentrations (2021–2023) in Omkoi (rural) and San Pa Tong (peri-urban) districts. Data are presented as mean ± standard deviation.

Sensor calibration, data averaging, and quality control procedures followed standardized protocols established by RIHES. Annual mean concentrations of PM_2.5_ were calculated from three-year averaged data (2021–2023) and are presented in Figure 2. Omkoi District reported an annual mean PM_2.5_ concentration of 15.58 ± 5.60 µg/m^3^, while San Pa Tong District recorded a value of 19.27 ± 7.32 µg/m^3^. Both districts exceeded the annual PM_2.5_ safety threshold of 15 µg/m^3^ set by the National Environment Board of Thailand [30].

### 2.3. Study Subjects

Participants were recruited based on specific inclusion and exclusion criteria. Eligible individuals included men or women aged 35 years or older who had been residing continuously in either Omkoi or San Pa Tong District for at least three years without any relocation during that period. Participants were required to have a clinical diagnosis of hypertension or exhibit a systolic blood pressure (SBP) ≥ 140 mmHg and/or diastolic blood pressure (DBP) ≥ 90 mmHg at the time of screening. The exclusion criteria included individuals diagnosed with any severe medical conditions, including cardiovascular diseases, chronic kidney disease, active infections, recent surgery, liver disease, gout, cancer, or hereditary disorders (e.g., thalassemia). Additionally, expectant or lactating women, as well as individuals with substance abuse, alcohol dependence, or psychiatric disorders that could potentially impede participation, were excluded. All participants received detailed information about the study objectives and procedures and provided written informed consent prior to any data collection or biological sample acquisition.

### 2.4. Study Design

This study employed a cross-sectional pilot study design to evaluate differences in lifestyle factors, including smoking habits, alcohol consumption, and dietary intake, and their associations with inflammation-related health outcomes among individuals with hypertension residing in rural and peri-urban areas exposed to PM_2.5_ pollution in Chiang Mai Province, Thailand.

Data collection was conducted in June 2024 in Omkoi District (representing the rural area) and San Pa Tong District (representing the peri-urban area). Information was obtained through a structured questionnaire, alongside physical assessments, including body weight, height, waist circumference, hip circumference, and blood pressure measurements. Blood samples were collected to assess fasting blood glucose, lipid profiles, liver and kidney function tests, and electrolyte levels at the Associated Medical Sciences Clinical Service Center, Faculty of Associated Medical Sciences, Chiang Mai University. A portion of each blood sample was centrifuged to separate serum, which was stored at −80 °C until analysis for vascular inflammation markers.

### 2.5. Assessment of Vascular Inflammation Markers

Serum concentrations of vascular inflammation markers, ICAM-1, VCAM-1, and IL-6, were quantified using enzyme-linked immunosorbent assay (ELISA) kits based on a colorimetric detection method. The following ELISA pair sets were used: Human ICAM-1 ELISA Pair Set (Cat. No. SEKA10346), Human VCAM-1 ELISA Pair Set (Cat. No. SEK10113), and Human IL-6 ELISA Pair Set (Cat. No. SEKB10395), all obtained from Sino Biological Inc. (Wayne, PA, USA). Optical density was measured at 450 nm using a microplate reader (BMG Labtech, Ortenberg, Germany). Assays were performed in duplicate, and marker concentrations were calculated from standard curves using recombinant protein standards. Results were expressed in pg/mL or ng/mL, as appropriate.

### 2.6. Assessment of Nutrient Intake

Dietary intake data were collected using three consecutive 24 h dietary recalls, consisting of two weekdays and one weekend day. One week prior to data collection, participants were informed and encouraged to record or photograph all foods and beverages consumed to minimize recall bias. During the collection period, trained research staff conducted face-to-face interviews using a structured questionnaire and visual aids, including plates, bowls, measuring cups, and spoons, to improve portion size estimation. Energy and nutrient intakes were analyzed using INMUCAL–Nutrients Version 4.0, a dietary analysis software developed by the Institute of Nutrition, Mahidol University, Thailand. Daily intake values were calculated and presented as average daily consumption per participant.

### 2.7. Statistical Analysis

All statistical analyses were performed using SPSS software, version 15.0 (SPSS Inc., Chicago, IL, USA). Descriptive statistics were used to summarize participant characteristics. Continuous variables were presented as means ± standard deviations (SD), while categorical variables were reported as counts (n) and percentages (%). Categorical variables such as demographic characteristics, lifestyle factors, and health status (sex, age group, smoking status, alcohol consumption, and presence of chronic diseases) were compared between rural and peri-urban groups using the Chi-square test. For continuous variables (anthropometric measures, blood biochemical parameters, and dietary intake), group comparisons were performed using the Mann–Whitney U test, due to non-normal data distribution. A *p*-value < 0.05 was considered statistically significant. To examine the associations between lifestyle behaviors (smoking, alcohol consumption) and nutrient intakes with serum inflammatory biomarkers (ICAM-1, VCAM-1, IL-6), partial correlation analysis was conducted, adjusting for covariates. To account for multiple comparisons and control the false discovery rate, *p*-values obtained from the partial correlation analyses were adjusted using the Benjamini–Hochberg correction. This method ranks all *p*-values in ascending order and compares each with an adjusted significance threshold calculated using the formula:

(1) $$Threshold=\frac{i}{n}\;\times \alpha$$

where *i* is the rank of the *p*-value (1 = smallest, *n* = largest), *n* is the total number of tests performed, and *α* is the desired false discovery rate (set at 0.05 in this study). A *p*-value is considered statistically significant if it is less than or equal to its corresponding threshold.

## 3. Results

### 3.1. Baseline Characteristics of Participants in Rural and Peri-Urban Areas

The baseline characteristics of participants who reside in rural and peri-urban areas are presented in Table 1. A total of 47 participants were enrolled, with 23 originating from rural areas and 24 from peri-urban areas. The proportion of females in peri-urban areas was considerably higher than that in rural areas (87.5% vs. 60.9%, *p* = 0.036), with the majority being female (74.5%). The mean age of the participants was 57.7 ± 8.1 years, with those residing in peri-urban areas being marginally older than their rural counterparts (60.0 ± 9.4 vs. 55.4 ± 5.8 years). However, this difference was not statistically significant (*p* = 0.053).

In terms of alcohol consumption, a greater proportion of participants in the rural group reported drinking alcohol compared to those in the peri-urban group (60.9% vs. 34.8%, respectively). However, this difference was not statistically significant (*p* = 0.077). Regarding smoking status, a significantly higher proportion of peri-urban participants had never smoked compared to rural participants (78.3% vs. 39.1%, *p* = 0.012). Among chronic diseases, the prevalence of diabetes mellitus and hyperlipidemia did not differ significantly between rural and peri-urban areas. All participants were diagnosed with hypertension by design, and no cases of stroke or heart disease were reported. Other comorbidities (GERD, allergy/asthma, herniated disk, thyroid disorders) were present in 10.6% of the participants, with no significant difference between groups (*p* = 0.348).

### 3.2. Physical Examination Parameters of Participants in Rural and Peri-Urban Areas

The physical examination parameters of participants who reside in rural and peri-urban areas are presented in Table 2. In terms of body height, body weight, body mass index (BMI), waist circumference (WC), SBP, DBP, and heart rate, no significant differences were observed between the groups. Nevertheless, a statistically significant difference was observed in the waist-to-hip ratio (WHR). The rural group exhibited a higher mean WHR than the peri-urban group (0.90 ± 0.07 vs. 0.85 ± 0.07, *p* = 0.010). The WHR of females in the rural group was substantially higher than that of females in the peri-urban group when stratified by sex (0.90 ± 0.07 vs. 0.84 ± 0.06, *p* = 0.043), while no significant difference was observed among males.

### 3.3. Blood Chemistry Parameters of Participants in Rural and Peri-Urban Areas

The blood chemistry parameters of participants who reside in rural and peri-urban areas are shown in Table 3. Fasting blood glucose (FBG), total cholesterol (TC), low-density lipoprotein cholesterol (LDL-C), kidney function markers (creatinine and blood urea nitrogen), and serum electrolytes (calcium, sodium, potassium, chloride, and carbon dioxide) did not exhibit any significant differences between the two groups. The rural group exhibited higher triglyceride (TG) levels than the peri-urban group (137.0 ± 55.5 vs. 107.8 ± 45.3 mg/dL), but this difference did not reach statistical significance (*p* = 0.057). On the other hand, the peri-urban group exhibited substantially higher levels of high-density lipoprotein cholesterol (HDL-C) than the rural group (48.6 ± 11.4 vs. 42.6 ± 12.4 mg/dL, *p* = 0.025).

For liver function tests, levels of alanine aminotransferase (ALT), aspartate aminotransferase (AST), and alkaline phosphatase (ALP) were significantly elevated in the rural group compared to the peri-urban group. Specifically, ALT was 18.2 ± 12.1 U/L in the rural group versus 12.3 ± 14.4 U/L in the peri-urban group (*p* = 0.007); AST was 30.3 ± 18.2 vs. 26.6 ± 31.2 U/L (*p* = 0.008); and ALP was 116.4 ± 28.9 vs. 89.8 ± 26.3 U/L (*p* = 0.002), respectively.

### 3.4. Vascular Inflammatory Biomarkers of Participants in Rural and Peri-Urban Areas

**Figure 3:**
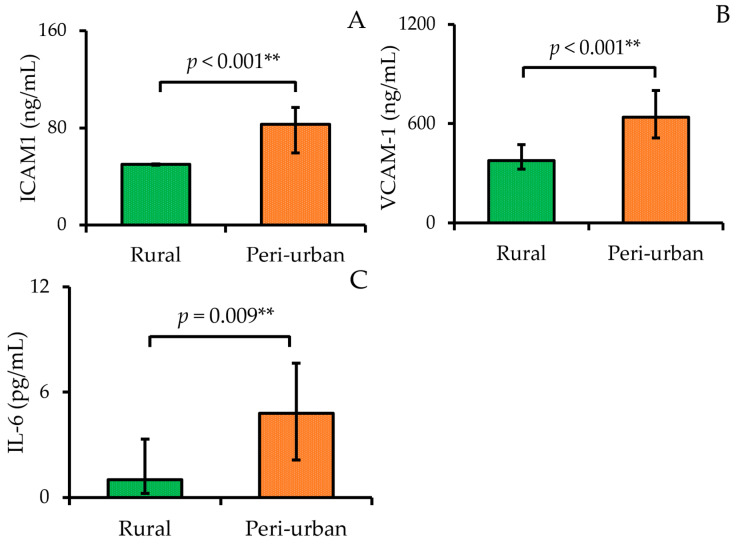
Median serum concentrations and interquartile ranges (Q1–Q3) of (**A**) ICAM-1, (**B**) VCAM-1, and (**C**) IL-6 among participants residing in rural (n = 23) and peri-urban (n = 24) areas. Data are presented as median (Q1–Q3) and compared using the Mann–Whitney U test. ** indicates statistically significant results at *p* < 0.01.

The serum concentrations of vascular inflammatory biomarkers (ICAM-1, VCAM-1, and IL-6) among participants residing in rural and peri-urban areas are shown in Figure 3. The peri-urban group exhibited significantly higher IL-6 levels, with a median of 4.80 pg/mL (2.13–7.66), compared to 1.02 pg/mL (0.24–3.33) in the rural group (*p* = 0.009). Regarding endothelial adhesion molecules, ICAM-1 concentrations were elevated in the peri-urban group, with a median of 83.00 ng/mL (59.34–96.94), while the rural group had a significantly lower median of 50.08 ng/mL (49.36–50.52) (*p* < 0.001). Similarly, serum VCAM-1 levels were considerably higher in peri-urban participants [median: 639.29 ng/mL (512.40–799.40)] than in rural participants [median: 376.50 ng/mL (324.83–473.17)], with a statistically significant difference (*p* < 0.001).

### 3.5. Nutrient Intake Among Participants in Rural and Peri-Urban Areas

The macronutrient intake distribution as a percentage of total energy among participants in rural and peri-urban areas is depicted in Table 4. The rural group had a higher median carbohydrate intake [60.1% (49.9–72.1)] compared to the peri-urban group [55.9% (53.2–67.3)]; however, the difference was not statistically significant (*p* = 0.338). Median protein intake was comparable between groups, with rural participants consuming 15.9% (12.8–22.1) of total energy and peri-urban participants consuming 16.8% (14.1–23.4) (*p* = 0.848). Fat intake tended to be slightly higher among peri-urban individuals [22.8% (17.3–28.9)] than rural individuals [20.2% (8.4–27.3)], but this difference also did not reach statistical significance (*p* = 0.194).

The daily intake of energy and nutrients among participants residing in rural and peri-urban areas is presented in Table 5. There were no significant differences between the two groups in total energy intake, macronutrients (carbohydrates, proteins, fats), or several key minerals, including calcium, iron, sodium, and phosphorus (all *p* > 0.05). However, significant differences were observed for several nutrients. Peri-urban participants had significantly higher sugar intake than those in rural areas [median: 39.3 g (22.7–58.1) vs. 19.0 g (12.8–33.4), *p* = 0.004]. In contrast, rural participants consumed significantly more vegetable protein [29.1 g (22.6–34.4) vs. 22.8 g (16.3–29.7), *p* = 0.043] and cholesterol [275.8 mg (182.6–481.6) vs. 169.2 mg (83.2–266.4), *p* = 0.023] compared to their peri-urban counterparts.

Regarding micronutrient intake, rural participants demonstrated significantly higher intake of selenium [63.3 µg (28.9–95.3) vs. 25.4 µg (6.0–48.1), *p* = 0.011], vitamin A [429.4 µg RAE (308.1–1006.2) vs. 234.1 µg RAE (88.7–348.2), *p* = 0.005], and β-carotene [1598.9 µg (853.8–2426.8) vs. 744.3 µg (287.1–1853.5), *p* = 0.016]. Vitamin C intake was also significantly higher in the rural group [96.3 mg (51.5–156.5)] compared to the peri-urban group [47.1 mg (26.6–80.6), *p* = 0.002]. No statistically significant differences were found for other micronutrients such as calcium, iron, sodium, phosphorus, magnesium, copper, zinc, retinol, and the B-complex vitamins (B1, B2, and B12).

### 3.6. Associations Between Lifestyle Behaviors and Vascular Inflammatory Biomarkers

Partial correlation analyses were conducted to explore the associations between lifestyle behaviors, dietary nutrient intakes, and serum concentrations of vascular inflammatory biomarkers (ICAM-1, VCAM-1, and IL-6) among participants, adjusting for sex, age, BMI, WHR, HDL-C, and total energy intake (Table 6). Alcohol consumption and smoking status were mutually adjusted, while nutrient intakes were additionally adjusted for both behaviors and log-transformed prior to analysis. Sugar intake was positively correlated with endothelial adhesion molecules, reaching statistical significance for ICAM-1 (*r* = 0.574, *p* < 0.001) and VCAM-1 (*r* = 0.533, *p* < 0.001). In contrast, higher intakes of selenium and vitamin C were inversely correlated with ICAM-1 levels, with significant associations observed for selenium (*r* = −0.473, *p* = 0.002) and vitamin C (*r* = −0.497, *p* = 0.001). Intake of selenium, vitamin B6, and vitamin C were also inversely associated with VCAM-1 levels, with significant relationships revealed for selenium (*r* = −0.485, *p* = 0.002), vitamin B6 (*r* = −0.552, *p* < 0.001), and vitamin C (*r* = −0.553, *p* < 0.001).

## 4. Discussion

Hypertension is one of the major risk factors for CVD, leading to disease development through endothelial dysfunction and chronic inflammation [2]. Lifestyle behaviors including smoking, drinking, and eating poorly are known to increase CVD risk [21]. Additionally, growing evidence has linked environmental exposures, particularly PM_2.5_, to vascular inflammation and adverse cardiovascular outcomes [13,14,15]. Given the interaction between behavioral and environmental factors, this study aimed to investigate differences in vascular inflammation among individuals with hypertension residing in rural and peri-urban areas of Chiang Mai Province, Thailand—regions affected by PM_2.5_ pollution.

The baseline characteristics of the participants revealed significant differences. While the mean age of peri-urban participants was slightly higher than their rural participants (60.0 ± 9.4 vs. 55.4 ± 5.8 years), this difference was not statistically significant (*p* = 0.053). However, age remains an important non-modifiable risk factor for CVD [31]. Several studies have demonstrated that vascular inflammation, arterial stiffness, and endothelial dysfunction increase with age, which collectively contribute to heightened CVD risk [32,33]. In particular, age-related changes in the immune system may promote a pro-inflammatory state, characterized by elevated cytokines such as IL-1β, IL-6, and TNF-α [34]. In terms of lifestyle behaviors, participants in the rural group reported a significantly higher rate of smoking (*p* = 0.012) and a non-significant trend toward higher alcohol consumption (*p* = 0.077). Excessive alcohol consumption and smoking are recognized CVD risk factors that enhance vascular injury by promoting systemic inflammation, oxidative stress, and lipid peroxidation [35,36,37]. According to a study by Kadri et al. (2021), tobacco use is one of the most powerful predictors of acute myocardial infarction [38]. Similarly, chronic alcohol consumption has been associated with increased levels of inflammatory cytokines, including IL-6, TNF-α, and IL-1β [39].

Anthropometric indicators such as body weight, BMI, WC, and WHR are key predictors of CVD risk [40]. Central obesity, often assessed through WC and WHR, is more strongly associated with metabolic and cardiovascular complications than general obesity measured by BMI alone [40,41,42]. Abdominal fat deposition is metabolically active and contributes to systemic inflammation, insulin resistance, and endothelial dysfunction, all of which enhance the atherosclerotic process [43]. In this study, although the overall BMI and WC values were not significantly different between rural and peri-urban groups, both groups exhibited mean values within the overweight range (BMI ≥ 23 kg/m^2^ for Asians), suggesting a moderate baseline risk of cardiometabolic complications [44]. Interestingly, WHR was higher among rural participants, particularly in females. This may be partially explained by the younger average age in the rural group, as several studies have suggested that younger individuals, particularly women in low-to-middle-income countries, may exhibit a higher WHR due to differing patterns of fat distribution, hormonal profiles, or postnatal body changes [45,46].

Blood lipid profiles are critical indicators of CVD risk, particularly in individuals with hypertension. Elevated TG levels are a known component of atherogenic dyslipidemia and are independently associated with increased CVD risk, especially when coexisting with low levels of HDL-C [47]. In the present study, participants in the rural area exhibited a trend toward higher TG levels (*p* = 0.057), while those in the peri-urban area had significantly higher HDL-C concentrations. These differences may reflect underlying lifestyle and dietary factors. For instance, elevated TG levels in rural participants could be linked to higher intake of carbohydrate-rich, low-quality diets, alcohol consumption, or reduced lipid metabolism efficiency, conditions that have been reported in rural populations [48]. Conversely, higher HDL-C levels observed among peri-urban participants may be associated with increased physical activity, which is known to increase HDL-C concentrations [49,50].

Furthermore, liver function tests (AST, ALT, and ALP) were significantly higher in rural participants. While the mean values remained within or near the upper normal range (AST: <35 U/L, ALT: 29–33 U/L for males and 19–25 U/L for females, ALP: 30–120 U/L) [51], elevated levels may indicate subclinical hepatic stress. This finding may be partly explained by the higher prevalence of alcohol consumption in the rural group, consistent with previous research linking chronic alcohol use to hepatocellular injury and elevation of aminotransferases [52,53]. Despite these biochemical differences, no significant variations were observed in kidney function (serum creatinine, BUN), or serum electrolytes between rural and peri-urban participants. This suggests that lipid and liver parameters may be more sensitive to lifestyle and behavioral differences, such as alcohol consumption or dietary composition.

In this study, we measured three key biomarkers—ICAM-1, VCAM-1, and IL-6—all of which are involved in mediating leukocyte adhesion, transmigration, and vascular inflammation [9,10]. Our findings revealed significantly lower levels of ICAM-1, VCAM-1, and IL-6 in rural participants compared to their peri-urban participants. These results suggest that rural participants, despite higher rates of smoking and alcohol use, may exhibit less endothelial activation, possibly reflecting protective lifestyle or dietary factors, such as greater intake of antioxidant-rich foods (β-carotene, selenium, and vitamin C) [54].

Regarding dietary intake, although total energy consumption and macronutrient distribution were similar between individuals with hypertension living in rural and peri-urban areas, notable differences were observed in specific dietary components relevant to cardiovascular and inflammatory risk. Participants from peri-urban areas consumed significantly higher amounts of sugar, which may be attributed to the greater availability and accessibility of sugar-sweetened beverages and confectioneries in urbanized environments. This trend reflects the influence of Western dietary culture that has expanded from urban centers into peri-urban communities, as evidenced by the increased consumption of items such as flavored coffee drinks and processed sweets (doughnuts and cakes) [25,55].

Participants residing in rural areas exhibited higher intakes of cholesterol, vegetable protein, selenium, vitamin A, β-carotene, vitamin B6, niacin, and vitamin C compared to those in peri-urban settings. This dietary pattern may reflect distinctive local food practices rooted in traditional rural lifestyles. For instance, eggs are commonly consumed as a dietary staple in rural communities and serve as a rich source of cholesterol, selenium, vitamin A (retinol), vitamin B6, and niacin [56,57]. In addition to animal-based foods, rural individuals frequently incorporate dark green leafy vegetables such as Chinese kale (*Brassica alboglabra*) and Chinese cabbage (*Brassica rapa*) into daily meals through stir-frying, boiling, or steaming. These vegetables are excellent sources of β-carotene (a provitamin A), vitamin C, and folate, and they also contribute modest amounts of niacin and vitamin B6 [58,59]. Moreover, fermented soybean products, such as ‘thua nao’, are widely used in rural Thai cuisine, often added to chili pastes (nam prik), curry dishes, and soups. Fermented soybeans are known to provide high-quality vegetable protein, as well as significant amounts of niacin, vitamin B6, and selenium [60,61,62]. These traditional practices may partially explain the observed higher intake of antioxidant-related micronutrients and plant-based protein among rural participants.

Partial correlation analysis, adjusted for age, sex, BMI, waist-to-hip ratio, HDL-C, total energy intake, alcohol consumption, and smoking status, revealed a significant positive association between sugar intake and the vascular inflammatory markers ICAM-1 and VCAM-1. These findings are consistent with prior studies suggesting that high dietary sugar promotes low-grade inflammation through increased oxidative stress, endothelial dysfunction, and activation of pro-inflammatory signaling pathways such as NF-κB and MAPK. Excessive sugar intake has also been linked to increased circulating adhesion molecules and cytokines, which play critical roles in the pathogenesis of atherosclerosis and hypertension [23,24]. These results may help explain the observed trend toward higher fasting blood glucose levels among peri-urban residents.

In contrast, inverse associations were observed between selenium and vitamin C with ICAM-1, as well as between selenium, vitamin B6, and vitamin C with VCAM-1. These findings support the potential protective roles of antioxidant and anti-inflammatory nutrients in modulating vascular inflammation. Selenium is a trace element essential for the function of selenoproteins, including glutathione peroxidases and thioredoxin reductases, which mitigate oxidative stress and inflammatory responses. Previous studies have shown that adequate selenium status is inversely associated with vascular inflammation and endothelial dysfunction [63,64]. Vitamin B6 has been reported to suppress vascular inflammation by regulating homocysteine metabolism and inhibiting the expression of adhesion molecules. Deficiency in vitamin B6 has been linked to elevated levels of pro-inflammatory cytokines and increased cardiovascular risk [65,66]. Vitamin C, a potent water-soluble antioxidant, has been shown to reduce oxidative damage, inhibit endothelial activation, and downregulate the expression of ICAM-1 and VCAM-1. Several studies have demonstrated that vitamin C supplementation can lower circulating adhesion molecules and improve vascular function, particularly in individuals with elevated oxidative stress or hypertension [67,68].

Interestingly, despite the higher prevalence of current smoking among rural participants and a comparable proportion of alcohol consumption between groups, partial correlation analysis revealed no significant associations between either smoking or alcohol intake and any of the vascular inflammatory biomarkers (ICAM-1, VCAM-1, or IL-6) after adjusting for relevant covariates. This finding contrasts with other studies that have linked chronic smoking and excessive alcohol consumption to increased systemic and vascular inflammation [35,36,37,38,39]. The effects of dietary antioxidants and other lifestyle factors may have mitigated the inflammatory impact of smoking and alcohol in this population.

However, a concerning finding from this study is the high sodium intake observed in both groups, exceeding 3000 mg/day, which is substantially above the recommended limit of 2000 mg/day set by the Thai Ministry of Public Health [69]. Elevated dietary sodium intake is a well-established contributor to hypertension and cardiovascular disease risk [70]. This highlights the urgent need for public health strategies aimed at reducing sodium consumption across both rural and peri-urban populations to mitigate future cardiovascular complications.

Although this study was conducted as a cross-sectional pilot study with a relatively small sample size, which constitutes a limitation, the observed associations between specific nutrient intakes and vascular inflammation are noteworthy. The findings suggest that dietary patterns rich in antioxidant related nutrients, particularly those characteristics of rural populations, may exert protective effects against endothelial inflammation. These dietary differences, rooted in traditional food practices, stand in contrast to the more sugar-dense and potentially pro-inflammatory diets observed in peri-urban settings. Given the exploratory nature of this study, further research involving larger, more diverse cohorts is essential to confirm these associations and establish the generalizability of the findings. Additionally, longitudinal studies would be valuable for assessing causal relationships between dietary exposures and inflammatory outcomes. Moreover, future research should incorporate biomarkers of PM_2.5_ exposure such as urinary 1-hydroxypyrene, 8-hydroxy-2′-deoxyguanosine (8-OHdG), or serum oxidative stress markers to verify whether the observed inflammatory responses are directly attributable to chronic exposure to airborne particulate matter [71]. The integration of dietary, behavioral, and environmental exposure data will provide a more comprehensive understanding of modifiable factors influencing cardiovascular risk among individuals living in PM_2.5_-polluted environments.

## 5. Conclusions

This pilot study suggests that rural dietary patterns, characterized by higher intakes of antioxidant-related nutrients, such as selenium, β-carotene, niacin, and vitamins A, B6, and C, may contribute to lower levels of vascular inflammation biomarkers (ICAM-1, VCAM-1, and IL-6) among individuals with hypertension living in PM_2.5_-exposed environments. Correlation analyses revealed that sugar intake was positively associated with ICAM-1 and VCAM-1, whereas selenium and vitamin C were inversely associated with both biomarkers, and vitamin B6 was inversely associated with VCAM-1 only. Although rural participants exhibited a higher prevalence of smoking, neither smoking nor alcohol consumption were significantly associated with inflammatory marker concentrations. These findings highlight the potential role of dietary composition in modulating vascular inflammation and emphasize the need for larger, longitudinal studies to confirm these associations and inform targeted nutritional strategies aimed at reducing cardiovascular risk in polluted environments.

## Figures and Tables

**Table 1:** Baseline characteristics of among participants who live in rural and peri-urban areas.

Characteristics	Rural (n = 23)	Peri-Urban (n = 24)	Total (n = 47)	*p*-Value
**Sex, N (%)**				0.036 *
Male	9 (39.1%)	3 (12.5%)	12 (25.5%)	
Female	14 (60.9%)	21 (87.5%)	35 (74.5%)	
**Age (years), mean ± SD**	55.4 ± 5.8	60.0 ± 9.4	57.7 ± 8.1	0.053
**Alcohol consumption, N (%)**				0.077
Never drank	9 (39.1%)	15 (65.2%)	24 (52.2%)	
Drank	14 (60.9%)	8 (34.8%)	22 (47.8%)	
No answer	-	1		
**Smoking status, N (%)**				0.012 *
Never smoked	9 (39.1%)	18 (78.3%)	27 (58.7%)	
Current smoker	8 (34.8%)	1 (4.4%)	9 (19.6%)	
Former smoker	6 (26.1%)	4 (17.4%)	10 (21.7%)	
**Chronic diseases, N (%)**				
Diabetes mellitus	5 (21.7%)	4 (16.7%)	9(19.2%)	0.724
Hypertension	20 (87.0%)	21 (87.5%)	41 (87.2%)	1.000
Hyperlipidemia	12 (52.2%)	17 (70.8%)	29 (61.7%)	0.188
Stroke	0	0	0	-
Heart disease	0	0	0	-
**Other diseases** (e.g., GERD, allergy/asthma, herniated disk, thyroid disorders)	1 (4.4%)	4 (16.7%)	5 (10.6%)	0.348

Data are presented as number and percentage, or mean ± standard deviation (SD). * indicates statistically significant difference at *p* < 0.05.

**Table 2:** Physical examination parameters of participants who live in rural and peri-urban areas.

Physical Examination	Rural (n = 23)	Peri-Urban (n = 24)	*p*-Value
Body height (cm)	155.4 ± 7.1	155.5 ± 5.5	0.972
Body weight (kg)	61.5 ± 12.1	60.7 ± 12.7	0.826
Body mass index, BMI (kg/m^2^)	25.4 ± 4.8	25.0 ± 4.8	0.787
Waist circumference, WC (cm)	87.4 ± 12.5	84.7 ± 11.0	0.446
Male	87.4 ± 9.2	92.3 ± 5.8	0.409
Female	87.4 ± 14.6	83.5 ± 11.2	0.389
Waist-to-hip ratio, WHR	0.90 ± 0.07	0.85 ± 0.07	0.010 *
Male	0.90 ± 0.05	0.90 ± 0.03	0.916
Female	0.90 ± 0.07	0.80 ± 0.06	0.043 *
Systolic blood pressure, SBP (mmHg)	134.3 ± 12.6	128.9 ± 14.5	0.182
Diastolic blood pressure, DBP (mmHg)	83.2 ± 8.6	81.3 ± 11.0	0.500
Heart rate (bpm)	76.9 ± 10.8	80.0 ± 15.1	0.602

Data are presented as mean ± standard deviation (SD). * indicates statistically significant difference at *p* < 0.05.

**Table 3:** Blood chemistry parameters of participants residing in rural and peri-urban areas.

Blood Chemistry Parameters	Rural (n = 23)	Peri-Urban (n = 24)	*p*-Value
Fasting blood glucose, FBG (mg/dL)	108.9 ± 40.9	111.1 ± 33.7	0.277
**Lipid profiles**			
Total cholesterol, TC (mg/dL)	197.9 ± 31.1	192.8 ± 37.8	0.618
Triglyceride, TG (mg/dL)	137.0 ± 55.5	107.8 ± 45.3	0.057
High-density lipoprotein cholesterol, HDL-C (mg/dL)	42.6 ± 12.4	48.6 ± 11.4	0.025 *
Low-density lipoprotein cholesterol, LDL-C (mg/dL)	144.9 ± 40.6	133.4 ± 45.3	0.367
**Liver function tests**			
Alanine aminotransferase, ALT (U/L)	18.2 ± 12.1	12.3 ± 14.4	0.007 **
Aspartate aminotransferase, AST (U/L)	30.3 ± 18.2	26.6 ± 31.2	0.008 **
Alkaline phosphatase, ALP (U/L)	116.4 ± 28.9	89.8 ± 26.3	0.002 **
**Kidney function tests**			
Creatinine (mg/dL)	0.8 ± 0.3	0.7 ± 0.2	0.133
Blood urea nitrogen, BUN (mg/dL)	12.8 ± 4.6	12.5 ± 2.8	0.730
**Serum electrolytes**			
Calcium, Ca (mg/dL)	9.1 ± 0.4	9.1 ± 0.5	0.571
Sodium, Na (mmol/L)	141.6 ± 8.4	142.8 ± 1.7	0.383
Potassium, K (mmol/L)	4.1 ± 0.4	4.0 ± 0.5	0.300
Chloride, Cl (mmol/L)	106.3 ± 2.7	105.9 ± 2.5	0.548
Carbon dioxide, CO_2_ (mmol/L)	24.6 ± 2.1	25.4 ± 2.4	0.217

Data are presented as mean ± standard deviation (SD). * indicates statistically significant difference at *p* < 0.05; ** indicates highly significant difference at *p* < 0.01.

**Table 4:** Distribution of macronutrient intake (% of total energy) among participants residing in rural and peri-urban areas.

Macronutrients	Rural (n = 23)	Peri-Urban (n = 24)	*p*-Value ^ns^
Carbohydrate (%)	60.1 (49.9–72.1)	55.9 (53.2–67.3)	0.338
Protein (%)	15.9 (12.8–22.1)	16.8 (14.1–23.4)	0.848
Fat (%)	20.2 (8.4–27.3)	22.8 (17.3–28.9)	0.194

Data are presented as median (Q1–Q3) and compared using the Mann–Whitney U test. ^ns^ indicates no statistically significant difference between groups.

**Table 5:** Daily intake of energy and nutrients among participants in rural and peri-urban areas.

Variables	Rural (n = 23)	Peri-Urban (n = 24)	*p*-Value
Energy (kcal)	1794.2 (1514.1–2271.3)	1576.6 (1468.2–2322.9)	0.702
Carbohydrates (g)	275.5 (201.2–366.1)	269.5 (210.7–364.6)	0.686
Sugars (g)	19.0 (12.8–33.4)	39.3 (22.7–58.1)	0.004 **
Proteins (g)	72.6 (57.6–111.5)	76.6 (64.1–93.5)	0.898
Animal protein (g)	39.9 (24.8–84.1)	31.3 (27.6–56.7)	0.444
Vegetable protein (g)	29.1 (22.6–34.4)	22.8 (16.3–29.7)	0.043 *
Fats (g)	45.1 (16.3–58.3)	41.6 (31.0–68.6)	0.670
Total saturated fatty acids (g)	11.0 (3.4–17.5)	13.5 (7.7–20.5)	0.115
Cholesterol (mg)	275.8 (182.6–481.6)	169.2 (83.2–266.4)	0.023 *
Calcium (mg)	483.3 (311.6–908.1)	566.2 (251.4–874.3)	0.718
Phosphorus (mg)	734.6 (609.4–132.4)	731.6 (613.5–907.1)	0.407
Iron (mg)	15.0 (11.2–19.6)	12.8 (9.5–18.1)	0.317
Potassium (mg)	1744.7 (1354.8–2665.5)	1760.9 (1367.9–2131.6)	0.766
Sodium (mg)	3465.6 (2854.5–4156.4)	3248.6 (2670.4–4280.7)	0.831
Magnesium (mg)	62.6 (22.0–104.5)	50.8 (27.5–111.8)	0.101
Copper (mg)	1.2 (0.8–1.4)	0.9 (0.6–1.2)	0.898
Selenium (μg)	63.3 (28.9–95.3)	25.4 (6.0–48.1)	0.011 *
Zinc (mg)	7.9 (5.3–8.0)	6.7 (4.5–8.9)	0.686
Vitamin A (μg RAE)	429.4 (308.1–1006.2)	234.1 (88.7–348.2)	0.005 **
Retinol (μg)	161.1 (9.2–345.6)	23.3 (4.6–162.0)	0.160
β-carotene (μg)	1598.9 (853.8–3426.8)	744.3 (287.5–1883.5)	0.015 *
Vitamin B1 (mg)	1.0 (0.7–3.2)	1.2 (0.8–1.8)	0.831
Vitamin B2 (mg)	1.4 (1.1–1.8)	1.1 (0.7–1.5)	0.089
Vitamin B6 (mg)	0.7 (0.4–1.4)	0.4 (0.2–0.8)	0.033 *
Vitamin B12 (mg)	1.2 (0.01–3.4)	0.7 (0.001–2.4)	0.265
Niacin (mg)	20.0 (16.6–29.0)	17.4 (14.3–21.0)	0.050 *
Vitamin C (mg)	96.3 (51.5–136.8)	47.5 (33.6–57.4)	0.006 **
Vitamin E (mg)	0.5 (0.2–1.6)	1.0 (0.3–1.8)	0.523
Dietary fiber (g)	15.5 (10.1–20.1)	12.4 (8.7–17.3)	0.142

Data are presented as median (Q1–Q3) and compared using the Mann–Whitney U test. * indicates statistically significant difference at *p* ≤ 0.05; ** indicates highly significant difference at *p* < 0.01.

**Table 6:** Partial correlation coefficients between lifestyle behaviors and vascular inflammatory biomarkers.

Lifestyle Behaviors	ICAM-1 ^a^		VCAM-1 ^a^		IL-6 ^a^	
	*r*	*p*-Value	*r*	*p*-Value	*r*	*p*-Value
**Alcohol consumption**	0.019	0.912	−0.081	0.912	−0.313	0.105
**Smoking status**	−0.210	0.206	−0.009	0.957	0.369	0.063
**Nutrient intakes ^a^**						
Sugars	0.574	**<0.001 ^1^**	0.533	**<0.001 ^4^**	0.374	0.045
Cholesterol	−0.157	0.340	−0.085	0.605	0.276	0.147
Selenium	−0.473	**0.002 ^7^**	−0.485	**0.002 ^6^**	−0.172	0.372
Vitamin A	−0.348	0.030	−0.362	0.024	−0.381	0.041
β-carotene	−0.235	0.150	−0.192	0.242	−0.424	0.022
Vitamin B6	−0.372	0.020	−0.552	**<0.001 ^3^**	−0.232	0.226
Niacin	−0.265	0.103	−0.334	0.038	−0.327	0.084
Vitamin C	−0.497	**0.001 ^5^**	−0.553	**<0.001 ^2^**	−0.163	0.397

Partial correlation coefficients (*r*) adjusted for sex, age, BMI, WHR, HDL-C, and energy intake (n = 47). Alcohol consumption and smoking were mutually adjusted. ^a^ nutrient intakes and vascular inflammatory biomarkers were log-transformed and adjusted for alcohol consumption, smoking, and covariates. Benjamini–Hochberg correction was applied (n = 30); bold values indicate significance at adjusted thresholds: ^1^ *p* < 0.0017, ^2^ *p* < 0.0033, ^3^ *p* < 0.0050, ^4^ *p* < 0.0067, ^5^ *p* < 0.0083, ^6^ *p* < 0.0100, and ^7^ *p* < 0.0117.

## Data Availability

The data presented in this study are available upon request from the corresponding author.

## Abbreviations

The following abbreviations are used in this manuscript:

H3K9me3	Trimethylation of lysine 9 on histone H3
H3K27me3	Trimethylation of lysine 27 on histone H3
CD40LG	CD40 ligand gene
lncRNA	Long non-coding RNA
NTAQHI	Northern Thailand Air Quality and Health Index
PEAMIR	PEA15-interacting microRNA regulatory lncRNA
ELISA	Enzyme-linked immunosorbent assay
RIHES	Research Institute for Health Sciences
8-OHdG	8-Hydroxydeoxyguanosine
ICAM-1	Intercellular adhesion molecule-1
LINE-1	Long interspersed nuclear element-1
VCAM-1	Vascular cell adhesion molecule-1
DASH	Dietary Approaches to Stop Hypertension
TNF-α	Tumor necrosis factor-alpha
HDL-C	High-density lipoprotein cholesterol
LDL-C	Low-density lipoprotein cholesterol
ACE	Angiotensin-converting enzyme
ALP	Alkaline phosphatase
ALT	Alanine aminotransferase
AST	Aspartate aminotransferase
BMI	Body mass index
BUN	Blood urea nitrogen
CRP	C-reactive protein
CVD	Cardiovascular disease
DBP	Diastolic blood pressure
FBG	Fasting blood glucose
HEC	Human Experimentation Committee
miR	MicroRNA
ROS	Reactive oxygen species
SBP	Systolic blood pressure
WHO	World Health Organization
WHR	Waist-to-hip ratio
NF-κB	Nuclear factor kappa-B
IL-6	Interleukin-6
PM_2.5_	Fine particulate matter
TG	triglyceride
TC	Total cholesterol
WC	Waist circumference

## References

[1] Gaidai O., Cao Y., Loginov S. (2023). Global Cardiovascular Diseases Death Rate Prediction. Curr. Probl. Cardiol..

[2] Prado A.F., Batista R.I.M., Tanus-Santos J.E., Gerlach R.F. (2021). Matrix Metalloproteinases and Arterial Hypertension: Role of Oxidative Stress and Nitric Oxide in Vascular Functional and Structural Alterations. Biomolecules.

[3] Cicalese S.M., da Silva J.F., Priviero F., Webb R.C., Eguchi S., Tostes R.C. (2021). Vascular Stress Signaling in Hypertension. Circ. Res..

[4] Wu O., Yuan C., Leng J., Zhang X., Liu W., Yang F., Zhang H., Li J., Khederzadeh S., Jiang Z. (2023). Colorable role of interleukin (IL)-6 in obesity hypertension: A hint from a Chinese adult case-control study. Cytokine.

[5] Fu M., Lv M., Guo J., Mei A., Qian H., Yang H., Wu W., Liu Z., Zhong J., Wei Y. (2025). The clinical significance of T-cell regulation in hypertension treatment. Front. Immunol..

[6] Smolgovsky S., Ibeh U., Tamayo T.P., Alcaide P. (2021). Adding insult to injury—Inflammation at the heart of cardiac fibrosis. Cell Signal..

[7] Bei Y.R., Zhang S.C., Song Y., Tang M.L., Zhang K.L., Jiang M., He R.C., Wu S.G., Liu X.H., Wu L.M. (2023). EPSTI1 promotes monocyte adhesion to endothelial cells in vitro via upregulating VCAM-1 and ICAM-1 expression. Acta Pharmacol. Sin..

[8] Taurone S., Santarelli M.T., De Santis E., Di Gioia C., Pompili E., Pellegrino F., Familiari P., Papa V., Zanza C., Coppola L. (2023). Porcine coronary arteries: Immunohistochemical profile of TNF-alpha, IL-1beta, TGF-beta1 and ICAM-1. Folia Morphol..

[9] Singh V., Kaur R., Kumari P., Pasricha C., Singh R. (2023). ICAM-1 and VCAM-1: Gatekeepers in various inflammatory and cardiovascular disorders. Clin. Chim. Acta.

[10] Bialecka M., Rac M., Dziedziejko V., Safranow K., Chlubek D., Rać M.E. (2024). An Evaluation of Plasma TNF, VEGF-A, and IL-6 Determination as a Risk Marker of Atherosclerotic Vascular Damage in Early-Onset CAD Patients. J. Clin. Med..

[11] Zhang S., Qian Z.M., Chen L., Zhao X., Cai M., Wang C., Zou H., Wu Y., Zhang Z., Li H. (2023). Exposure to Air Pollution during Pre-Hypertension and Subsequent Hypertension, Cardiovascular Disease, and Death: A Trajectory Analysis of the UK Biobank Cohort. Environ. Health Perspect..

[12] Qin P., Luo X., Zeng Y., Zhang Y., Li Y., Wu Y., Han M., Qie R., Wu X., Liu D. (2021). Long-term association of ambient air pollution and hypertension in adults and in children: A systematic review and meta-analysis. Sci. Total Environ..

[13] de Bont J., Jaganathan S., Dahlquist M., Persson Å., Stafoggia M., Ljungman P. (2022). Ambient air pollution and cardiovascular diseases: An umbrella review of systematic reviews and meta-analyses. J. Intern. Med..

[14] Chanda F., Lin K.X., Chaurembo A.I., Huang J.Y., Zhang H.J., Deng W.H., Xu Y.J., Li Y., Fu L.D., Cui H.D. (2024). PM_2.5_-mediated cardiovascular disease in aging: Cardiometabolic risks, molecular mechanisms and potential interventions. Sci. Total Environ..

[15] Ding R., Huang L., Yan K., Sun Z., Duan J. (2024). New insight into air pollution-related cardiovascular disease: An adverse outcome pathway framework of PM2.5-associated vascular calcification. Cardiovasc. Res..

[16] Parasin N., Amnuaylojaroen T. (2024). Effect of PM2.5 on burden of mortality from non-communicable diseases in northern Thailand. PeerJ.

[17] Supasri T., Gheewala S.H., Macatangay R., Chakpor A., Sedpho S. (2023). Association between ambient air particulate matter and human health impacts in northern Thailand. Sci. Rep..

[18] Zhao T., Qi W., Yang P., Yang L., Shi Y., Zhou L., Ye L. (2021). Mechanisms of cardiovascular toxicity induced by PM_2.5_: A review. Environ. Sci. Pollut. Res. Int..

[19] Gao Y., Zhang Q., Sun J., Liang Y., Zhang M., Zhao M., Zhang K., Dong C., Ma Q., Liu W. (2022). Extracellular vesicles derived from PM2.5-exposed alveolar epithelial cells mediate endothelial adhesion and atherosclerosis in ApoE^-/-^ mice. FASEB J..

[20] Simo L. (2024). The effects of PM2.5 air pollution on human health: A narrative review with a focus on cerebrovascular diseases. Environ. Dis..

[21] Liang Y., Liu F., Yin H., Shi X., Chen Y., Wang H., Wang Y., Bai B., Liu Y., Liu Q. (2023). Trends in unhealthy lifestyle factors in US NHANES respondents with cardiovascular disease for the period between 1999 and 2018. Front. Cardiovasc. Med..

[22] Diab A., Dastmalchi L.N., Gulati M., Michos E.D. (2023). A Heart-Healthy Diet for Cardiovascular Disease Prevention: Where Are We Now?. Vasc. Health Risk Manag..

[23] Pant N., Aasuri N., Shaikh M.A. (2024). Impact of Modern Food Style on Cardiovascular Health in Young Adults. Arch. Med. Rep..

[24] Angelico F., Baratta F., Coronati M., Ferro D., Del Ben M. (2023). Diet and metabolic syndrome: A narrative review. Intern. Emerg. Med..

[25] Jayasinghe S., Byrne N.M., Hills A.P. (2025). Cultural influences on dietary choices. Prog. Cardiovasc. Dis..

[26] Aleksandrova K., Koelman L., Rodrigues C.E. (2021). Dietary patterns and biomarkers of oxidative stress and inflammation: A systematic review of observational and intervention studies. Redox Biol..

[27] Kaur P., Dahiya R., Buttar H.S., Wilson D.W., De Meester F., Telessy I.G., Shah A.K., Tappia P.S., Dhalla N.S. (2024). Antiatherogenic Effects of Vitamins, Mediterranean Diet and DASH Diet: An Overview for the Prevention of Cardiovascular Diseases. Hydrophilic Vitamins in Health and Disease. Advances in Biochemistry in Health and Disease.

[28] Siviroj P., Wungrath J., Ongprasert K. (2024). Associated Factors of Dietary Patterns among Adolescents in the Rural Northern Region of Thailand: A Community-Based Cross-Sectional Study. Healthcare.

[29] Sachdev M., Misra A. (2023). Heterogeneity of Dietary practices in India: Current status and implications for the prevention and control of type 2 diabetes. Eur. J. Clin. Nutr..

[30] Pirard C., Charoenpanwutikul A. (2023). Comprehensive review of the annual haze episode in Northern Thailand. arXiv.

[31] Dhingra R., Vasan R.S. (2012). Age as a risk factor. Med. Clin. N. Am..

[32] Pacinella G., Ciaccio A.M., Tuttolomondo A. (2022). Endothelial Dysfunction and Chronic Inflammation: The Cornerstones of Vascular Alterations in Age-Related Diseases. Int. J. Mol. Sci..

[33] Clayton Z.S., Hutton D.A., Brunt V.E., VanDongen N.S., Ziemba B.P., Casso A.G., Greenberg N.T., Mercer A.N., Rossman M.J., Campisi J. (2021). Apigenin restores endothelial function by ameliorating oxidative stress, reverses aortic stiffening, and mitigates vascular inflammation with aging. Am. J. Physiol. Heart Circ. Physiol..

[34] Tylutka A., Walas Ł., Zembron-Lacny A. (2024). Level of IL-6, TNF, and IL-1β and age-related diseases: A systematic review and meta-analysis. Front. Immunol..

[35] Jin S., Kang P.M. (2024). A Systematic Review on Advances in Management of Oxidative Stress-Associated Cardiovascular Diseases. Antioxidants.

[36] Zhang X., Liu Y., Li S., Lichtenstein A.H., Chen S., Na M., Veldheer S., Xing A., Wang Y., Wu S. (2021). Alcohol consumption and risk of cardiovascular disease, cancer and mortality: A prospective cohort study. Nutr. J..

[37] Lopez-Jaramillo P., Joseph P., Lopez-Lopez J.P., Lanas F., Avezum A., Diaz R., Camacho P.A., Seron P., Oliveira G., Orlandini A. (2022). Risk factors, cardiovascular disease, and mortality in South America: A PURE substudy. Eur. Heart J..

[38] Kadri A.N., Khodor S., Ali A., Nusairat L., Mahmood A., Nahhas G., Dabbous S., Spears J., Jafri S., Werns S. (2021). National Trends of Tobacco, Alcohol, and Drug Use in Patients Admitted with Acute Myocardial Infarction. Cardiovasc. Revascularization Med..

[39] Dukić M., Radonjić T., Jovanović I., Zdravković M., Todorović Z., Kraišnik N., Aranđelović B., Mandić O., Popadić V., Nikolić N. (2023). Alcohol, Inflammation, and Microbiota in Alcoholic Liver Disease. Int. J. Mol. Sci..

[40] Xue R., Li Q., Geng Y., Wang H., Wang F., Zhang S. (2021). Abdominal obesity and risk of CVD: A dose-response meta-analysis of thirty-one prospective studies. Br. J. Nutr..

[41] Moltrer M., Pala L., Cosentino C., Mannucci E., Rotella C.M., Cresci B. (2022). Body mass index (BMI), waist circumference (WC), waist-to-height ratio (WHtR) e waist body mass index (wBMI): Which is better?. Endocrine.

[42] Ke J.F., Wang J.W., Lu J.X., Zhang Z.H., Liu Y., Li L.X. (2022). Waist-to-height ratio has a stronger association with cardiovascular risks than waist circumference, waist-hip ratio and body mass index in type 2 diabetes. Diabetes Res. Clin. Pract..

[43] Marketou M.E., Buechler N.S., Fragkiadakis K., Plevritaki A., Zervakis S., Maragkoudakis S., Tsiavos A., Simantirakis E., Kochiadakis G. (2023). Visceral fat and cardiometabolic future in children and adolescents: A critical update. Pediatr. Res..

[44] Sucato V., Coppola G., Manno G., Vadalà G., Novo G., Corrado E., Galassi A.R. (2023). Coronary Artery Disease in South Asian Patients: Cardiovascular Risk Factors, Pathogenesis and Treatments. Curr. Probl. Cardiol..

[45] Butovskaya M., Sorokowska A., Karwowski M., Sabiniewicz A., Fedenok J., Dronova D., Negasheva M., Selivanova E., Sorokowski P. (2017). Waist-to-hip ratio, body-mass index, age and number of children in seven traditional societies. Sci. Rep..

[46] Poobalan A., Aucott L. (2016). Obesity Among Young Adults in Developing Countries: A Systematic Overview. Curr. Obes. Rep..

[47] Kosmas C.E., Rodriguez Polanco S., Bousvarou M.D., Papakonstantinou E.J., Peña Genao E., Guzman E., Kostara C.E. (2023). The Triglyceride/High-Density Lipoprotein Cholesterol (TG/HDL-C) Ratio as a Risk Marker for Metabolic Syndrome and Cardiovascular Disease. Diagnostics.

[48] Gregory C.O., Dai J., Ramirez-Zea M., Stein A.D. (2007). Occupation is more important than rural or urban residence in explaining the prevalence of metabolic and cardiovascular disease risk in Guatemalan adults. J. Nutr..

[49] Ahn N., Kim K. (2016). High-density lipoprotein cholesterol (HDL-C) in cardiovascular disease: Effect of exercise training. Integr. Med. Res..

[50] Lazo-Porras M., Bernabe-Ortiz A., Málaga G., Gilman R.H., Acuña-Villaorduña A., Cardenas-Montero D., Smeeth L., Miranda J.J. (2016). Low HDL cholesterol as a cardiovascular risk factor in rural, urban, and rural-urban migrants: PERU MIGRANT cohort study. Atherosclerosis.

[51] Kalas M.A., Chavez L., Leon M., Taweesedt P.T., Surani S. (2021). Abnormal liver enzymes: A review for clinicians. World J. Hepatol..

[52] Singh G.P., Mitra Y., Singh J., Padda A. (2019). Prevalence of health problems among the regular alcohol users (chronic alcoholics) in urban and rural area of district Amritsar: Punjab: India. Public health Rev. Int. J. Public Health Res..

[53] Gedam S.R., Ajab D., Patil P.S., Sharma A., Kumar K., Babar V. (2019). Psychiatric Comorbidity, Severity of Dependence and Liver Enzymes Dysfunction among Alcohol Dependent Individuals: A Cross-sectional Study from Central Rural India. J. Clin. Diagn. Res..

[54] Giurranna E., Nencini F., Bettiol A., Borghi S., Argento F.R., Emmi G., Silvestri E., Taddei N., Fiorillo C., Becatti M. (2024). Dietary Antioxidants and Natural Compounds in Preventing Thrombosis and Cardiovascular Disease. Int. J. Mol. Sci..

[55] Seubsman S.A., Kelly M., Yuthapornpinit P., Sleigh A. (2009). Cultural resistance to fast-food consumption? A study of youth in North Eastern Thailand. Int. J. Consum. Stud..

[56] Cherian G., Watson R.R. (2009). Chapter 16—Eggs and Health: Nutrient Sources and Supplement Carriers. Complementary and Alternative Therapies and the Aging Population.

[57] Nimalaratne C., Wu J. (2015). Hen Egg as an Antioxidant Food Commodity: A Review. Nutrients.

[58] Gupta S., Gowri B.S., Lakshmi A.J., Prakash J. (2013). Retention of nutrients in green leafy vegetables on dehydration. J. Food Sci. Technol..

[59] Ebert A.W. (2022). Sprouts and Microgreens-Novel Food Sources for Healthy Diets. Plants.

[60] Jang Y., Kim M., Park J., Kim M. (2022). Evaluation of the thiamine, riboflavin, and niacin contents in fermented soybean processed foods in various Korean provinces. J. Korean Soc. Food Sci. Nutr..

[61] Knez E., Kadac-Czapska K., Grembecka M. (2023). Effect of Fermentation on the Nutritional Quality of the Selected Vegetables and Legumes and Their Health Effects. Life.

[62] Trevanich S., Sribuathong S., Bundidamorn D., Kristbergsson K., Ötles S. (2016). The Potential Health Benefits of Traditional Thai-Fermented Foods and Beverages. Functional Properties of Traditional Foods.

[63] Kuria A., Tian H., Li M., Wang Y., Aaseth J.O., Zang J., Cao Y. (2021). Selenium status in the body and cardiovascular disease: A systematic review and meta-analysis. Crit. Rev. Food Sci. Nutr..

[64] Giacconi R., Chiodi L., Boccoli G., Costarelli L., Piacenza F., Provinciali M., Malavolta M. (2021). Reduced levels of plasma selenium are associated with increased inflammation and cardiovascular disease in an Italian elderly population. Exp. Gerontol..

[65] Lotto V., Choi S.W., Friso S. (2011). Vitamin B6: A challenging link between nutrition and inflammation in CVD. Br. J. Nutr..

[66] Minović I., Kieneker L.M., Gansevoort R.T., Eggersdorfer M., Touw D.J., Voerman A.J., Connelly M.A., Boer R.A., Hak E., Bos J. (2020). Vitamin B6, Inflammation, and Cardiovascular Outcome in a Population-Based Cohort: The Prevention of Renal and Vascular End-Stage Disease (PREVEND) Study. Nutrients.

[67] Hernofialdi, Rini E.A., Machmud R. (2013). The effect of vitamin c supplementation on intercellular adhesion molecule-1 (ICAM-1) concentration on male adolescent obesity in Padang. Int. J. Pediatr. Endocrinol..

[68] Zheng R., Wanglaoji G., Ye J., Chan K.I., Li C., Zhong Z. (2024). Anti-inflammatory effects of natural products from vitamin C-rich fruits: A comprehensive review. Food Front..

[69] Satheannoppakao W., Kasemsup R., Inthawong R., Chariyalertsak S., Sangthong R., Taneepanichskul S., Putwatana P., Kessomboon P., Aekplakorn W. (2013). Sodium intake and socio-demographic determinants of the non-compliance with daily sodium intake recommendations: Thai NHES IV. J. Med. Assoc. Thai.

[70] O’Donnell M., Mente A., Yusuf S. (2014). Evidence relating sodium intake to blood pressure and CVD. Curr. Cardiol. Rep..

[71] Sabir S., Hongsibsong S., Chuljerm H., Parklak W., Ounjaijean S., Fakfum P., Kausar S., Kulprachakarn K. (2025). Assessment of urinary oxidative stress biomarkers associated with fine particulate matter (PM2.5) exposure in Chiang Mai, Thailand. PeerJ.

